# The Role of Complement in Myocardial Infarction Reperfusion Injury: An Underappreciated Therapeutic Target

**DOI:** 10.3389/fcell.2020.606407

**Published:** 2020-12-23

**Authors:** Carl-Wilhelm Vogel

**Affiliations:** University of Hawaii Cancer Center and Department of Pathology, John A. Burns School of Medicine, University of Hawaii at Manoa, Honolulu, HI, United States

**Keywords:** myocardial infarction, reperfusion injury, complement, complement depletion, cobra venom factor, humanized cobra venom factor

## Abstract

This article reviews the pathogenetic role of the complement system in myocardial infarction reperfusion injury. The complement activation pathways involved in myocardial tissue injury are identified, as are the complement-derived effector molecules. The results of past anti-complement therapies are reviewed; as the more recent therapeutic concept of complement depletion with humanized CVF described.

## Introduction

### The Complement System

**FIGURE 1:**
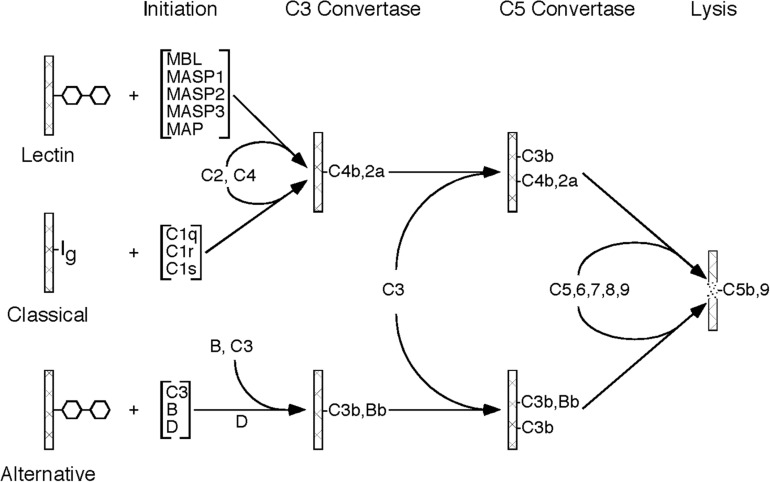
Schematic drawing showing the molecular organization of the three complement activation pathways (lectin, classical, alternative). Please note that all three pathways merge at the stage of C3 activation and, after forming a C5 convertase, initiate the formation of the C5b-9 complex (MAC).

The complement system is an important component of the immune system, with multiple roles in both the innate and adaptive immune response. The complement system is made up of approximately 20 plasma proteins and numerous receptors and regulatory proteins in the cell membranes of host cells. There are three pathways through which activation of the complement system can occur, referred to as classical pathway, lectin pathway, and alternative pathway, all of which share the same molecular architecture: an initial recognition event is amplified by a succession of proteolytic enzymes in a cascade-like fashion, merging at the step of C3 activation and resulting in the generation of multiple biologically active complement activation products (Figure 1).

### The Three Pathways of Complement Activation

The activation of the classical pathway usually involves binding of an antibody to its antigen with subsequent recognition by C1q. The alternative pathway is continuously activated in plasma at a slow rate. Activation is usually restricted by regulatory mechanisms, but activation can proceed on certain cell surfaces. Mostly, the alternative pathway activation loop enhances complement activation by the classical and lectin pathways. The lectin pathway is typically activated by mannose binding lectin (MBL) which recognizes certain carbohydrate structures rarely present on normal cells, but frequently present on pathogens and dying host cells.

### Complement Activation-Derived Effector Molecules

Complement activation by any of the three activation pathways leads to the generation of complement-derived effector molecules. Foremost, there are the two anaphylatoxins C3a and C5a. They are potent biologically active peptides released by the cleavage of C3 and C5, respectively, during complement activation through any of the three pathways. Both exhibit chemotaxis for important pro-inflammatory cells, and activate, in particular the strongly pro-inflammatory C5a, macrophages, eosinophils, and neutrophils. Carboxypeptidase N is the major regulator of anaphylatoxin activity, removing the C-terminal arginine residue from C3a to C5a. Both anaphylatoxins are rapidly inactivated by the removal of the C-terminal arginine residue. The resulting peptide after removal of the C-terminal arginine from C5a is referred to as C5a-des-arg. However, C5a-des-Arg still exhibits activity for neutrophils, making C5a a particularly powerful pro-inflammatory peptide.

Another important complement-derived effector is the macromolecular C5b-9 complex, also referred to as the membrane attack complex (MAC). It is generated by the cleavage of C5, with the activation product C5b serving as the nucleus for the assembly of the high molecular weight MAC, consisting of the complement proteins C5b, C6, C7, C8, and several C9. The MAC inserts itself into target membranes, leading to impairment of membrane function, physical destruction of targets cells, and ultimately cell death.

Another potent complement activation product is C3b. As the enzyme that cleaves C3 into C3a and C3b is typically located on a surface, nascent C3b can attach itself covalently to that surface through transesterification. The process of covalently binding of C3b molecules to a target (e.g., an immune complex, a microorganism, a cell), referred to as opsonization, has several biological consequences. C3b is recognized by the C3b receptor (CR1, CD35) on neutrophils, leading to cellular influx, nucleophile activation, inflammation, as well as ingestion and killing of target cells. Secondly, continuing deposition of C3b causes the creation of C5 convertases, leading to C5a generation and enhanced inflammation as well C5b generation and MAC formation as described above. Lastly, the covalently bound C3b is degraded in a step-wise fashion by Factor I to iC3b, C3dg, and eventually to C3d. The degradation products remain bound to their target surface. B cells express complement receptor 2 (CR2, CD21) which recognizes C3d. C3d bound to its antigen also binds to CR2 on follicular dendritic cells, eventually leading to antigen-specific IgG production.

This short overview of the complement system summarizes its important roles in host defense and immune response. Multiple excellent reviews of the complement system have been published (Walport, 2001a,b; Merle et al., 2015a,b).

### Complement, a Pathogenetic Factor in Many Diseases

Inappropriate complement activation leads damage of host cells and tissues. Accordingly, the complement system is also an important pathogenetic factor in numerous diseases including, but not limited to, rheumatoid arthritis, lupus erythematosus, myasthenia gravis, age-related macular degeneration (AMD), ischemia reperfusion injury, transplant rejection, paroxysmal nocturnal hemoglobinuria, (PNH), bullous pemphigoid, asthma, anti-phospholipid syndrome, autoimmune hemolytic anemia, and atypical hemolytic uremic syndrome (Vogel and Fritzinger, 2010; Vogel et al., 2014).

## The Role of Complement in Myocardial Reperfusion Injury

Complement activation has long been known to be an important factor for inflammation and injury of ischemic and infarcted myocardial tissue. The first report implicating the complement system demonstrated the generation of a C3-derived chemotactic activity resulting in intense accumulation of neutrophils in ischemic rat heart tissue (Hill and Ward, 1971) that could be suppressed by complement depletion with cobra venom factor (CVF) (Hill and Ward, 1971). Multiple authors demonstrated complement activation products such as C3d and C5b-9 in ischemic heart tissue (Mathey et al., 1994; Nijmeijer et al., 2004).

Reperfusion of infarcted myocardial tissue resulted, paradoxically, in significantly increased myocardial tissue damage of 50% or more (Ferreira, 2010), a phenomenon referred to as reperfusion injury. Several investigators demonstrated the significant role of complement activation in reperfusion injury of myocardial tissue in rats (MacLean et al., 1978), dogs (Maroko et al., 1978), and baboons (Pinckard et al., 1980). Complement depletion with CVF resulted in greatly reduced myocardial tissue damage and preservation of large areas of normal ventricular myocardium. The effect of complement depletion was demonstrated histologically, and by increased creatine phosphokinase (CPK) activity. There was also greatly reduced infiltration with neutrophils, and a virtual absence of deposited C3 in infarcted areas in CVF-treated animals. The extent of C3 and C5b-9 deposition is significantly increased in the myocardial tissue of humans after reperfusion (Nijmeijer et al., 2004).

### Complement Pathways Activated in Myocardial Reperfusion Injury

Of the three complement pathways, the most important one for inducing tissue damage in myocardial reperfusion injury is the lectin pathway (Jordan et al., 2001; Walsh et al., 2005; Panagiotou et al., 2018). Mice lacking MBL, and therefore a functional lectin pathway, do not develop cardiac reperfusion injury (Walsh et al., 2005); and an anti-MBL monoclonal antibody greatly reduced infarct size, C3 deposition, and neutrophil infiltration. There is also a role for the alternative pathway. Although there is no evidence that the alternative pathway is activated on altered surfaces in the myocardial tissue after ischemic damage, the alternative pathway has been shown to significantly contribute to the tissue damage by lectin pathway activation, as Factor B knock-out mice exhibited significantly reduced necrosis and diminished deposition of C3 (Chun et al., 2017). There has been some question about the role of the classical pathway in myocardial reperfusion injury (McMullen et al., 2006; Gorsuch et al., 2012). In other organ systems such as intestinal reperfusion, a role of the classical pathway has been described (Williams et al., 1999). In myocardial reperfusion injury, it has been shown that both MBL and natural IgM are required for complement activation (Busche et al., 2009). IgM appears to bind MBL, and leads to lectin pathway activation without involvement of C1q (Gorsuch et al., 2012). Mice deficient in B-cells, and therefore natural IgM, are protected from myocardial reperfusion injury (Zhang et al., 2006; Linfert et al., 2009). However, activation of the classical pathway by natural IgM antibodies to neoepitopes in injured heart tissue after ischemia cannot be excluded, as has been shown in other tissues(Narang et al., 2017).

### Complement Effector Molecules in Myocardial Reperfusion Injury

All complement-derived effector molecules as described above are involved in producing the inflammation, tissue injury, and necrosis of the heart tissue during reperfusion. C3 activation leads to covalent attachment of C3b to myocardial tissue. The concomitant release of the C3a anaphylatoxin causes influx and activation of neutrophils. C3b and its subsequent degradation products iC3b, C3dg, and C3d become covalently bound and durable signals of tissue inflammation. They are readily detected in infarcted tissue (Pinckard et al., 1980; Jordan et al., 2001; Stahl et al., 2003; Gorsuch et al., 2009). As a matter of fact, soluble radioactively labeled CR2 receptor (complement receptor type 2), recognizing the covalently bound C3d, was used to quantify the severity of myocardial tissue reperfusion injury (Sharif-Paghaleh et al., 2017).

Continued C3b deposition leads to the formation of C5 convertase activity. C5 activation leads to the release of the C5a anaphylatoxin with its strong pro-inflammatory activity for neutrophils. Moreover, C5 activation generates C5b and the subsequent formation of the membrane attack complex which can be detected in the injured myocardial tissue (Weisman et al., 1990; Mathey et al., 1994; Nijmeijer et al., 2004).

Activated neutrophils are important for myocardial inflammation and injury through release of reactive oxygen species (ROS) and pro-inflammatory cytokines. Activated neutrophils have also been shown to induce tissue damage in myocardial reperfusion injury through the formation of neutrophil extracellular traps (NETs) (Savchenko et al., 2014; Ge et al., 2015; Papayannopoulos, 2018). Whereas these neutrophil-derived cytotoxic activities are important mechanisms in myocardial reperfusion injury, they are only indirect consequences of complement activation.

## Anti-Complement Therapy in Myocardial Reperfusion

### Soluble Complement Receptor Type 1 (CR1)

Several studies using anti-complement therapeutics have been reported in animal models of myocardial reperfusion injury as well as in patients. In a rat model of myocardial reperfusion, treatment with a soluble form of complement receptor type 1 (CR1), a cofactor for C3b inactivation by Factor I, reduced the infarct size by 44% (Weisman et al., 1990). Similarly, a membrane-targeted version of soluble CR1 decreased infarct size and myocardial apoptosis, and resulted in an improved ejection fraction in pigs (Banz et al., 2007). However, clinical trials with soluble CR1 in cardiopulmonary bypass failed to meet clinical endpoints (Lazar et al., 2004; Li et al., 2004). Surprisingly, there was some therapeutic benefit seen in male patients, but not in female patients (Lazar et al., 2004).

### C1 Esterase Inhibitor

Another complement inhibitor that has been used in myocardial reperfusion injury is the C1 esterase inhibitor (C1INH). C1INH inhibits both the classical and lectin pathways of complement. Use of C1INH has shown beneficial effects in multiple animal models of myocardial reperfusion injury (Panagiotou et al., 2018). There have been a few studies in patients, well over a decade ago, with no impressive results (Panagiotou et al., 2018); and C1INH is currently not used in the treatment of myocardial reperfusion injury.

### Anti-C5 Antibodies

Antibodies to C5 have been employed in the therapy of myocardial reperfusion injury, based on the rationale that inhibition at the stage of C5 activation will prevent the generation of the pro-inflammatory C5a anaphylatoxin as well as the formation of the cytotoxic C5b-9 complex. Studies in animals (Vakeva et al., 1998; Pischke et al., 2017) and patients (Fitch et al., 1999; Granger et al., 2003; Armstrong et al., 2006) usually demonstrated beneficial effects, but failed to meet endpoints; and anti-C5 therapy is currently not in use to treat myocardial reperfusion injury. As appropriately pointed out, it is important to inhibit complement activation as far upstream as possible (Panagiotou et al., 2018).

### Complement Depletion With Humanized CVF (hCVF)

Cobra venom factor (CVF) is a structural and functional analog of complement component C3 (Fritzinger et al., 2009; Vogel and Fritzinger, 2010, 2017; Vogel et al., 2020). CVF forms a bimolecular enzyme with Factor B that is resistant to inactivation, leading to continuous C3 cleavage and complement depletion. As mentioned above, CVF was instrumental 40 years ago demonstrating the important role of complement in myocardial infarction reperfusion injury (MacLean et al., 1978; Maroko et al., 1978; Pinckard et al., 1980). Similarly, CVF had been used in animals, from mice to baboons, for over 50 years to demonstrate the role of complement in many diseases. CVF was never used in patients because CVF is immunogenic, and cobra venom is obviously an impractical source for a therapeutic agent. But we know that CVF depletes humans of their complement just like other mammals. Studies in cobra bite victims demonstrated complement depletion mirroring depletion in animals with CVF, with no indication of depletion-related toxicity (Warrell et al., 1976; Vogel and Fritzinger, 2017). Although recombinant CVF became available (Kock et al., 2004; Vogel et al., 2004), its immunogenicity prevented clinical application.

Given the high degree of structural homology between human C3 and CVF, humanized CVF was created by exchanging about 10% at the C-terminal end of human C3 with the homologous sequences from CVF. And even in this C-terminal end, approximately 43% of amino acid residues are identical to human C3. Accordingly, these humanized CVF molecules are human C3 derivatives in which only less than 6% of all amino acid residues differ from human C3. Moreover, the three-dimensional structure at the C-terminal end of humanized CVF is essentially indistinguishable from human C3 (Vogel et al., 2014; Vogel and Fritzinger, 2017). Humanized CVF exhibits complement depletion activity just like CVF in serum from multiple species, including human. Its complement depletion activity has also been shown *in vivo* in multiple species, from mouse to non-human primates (Vogel and Fritzinger, 2010). Moreover, for reasons not understood, humanized CVF only leads to C3 activation but not C5 activation, thereby not releasing the pro-inflammatory C5a anaphylatoxin.

Humanized CVF was able to greatly reduce the tissue damage in multiple murine models of disease with complement pathology (Vogel et al., 2014). Significantly, no toxicity has been observed with complement depletion with humanized CVF, including non-human primates (Vogel and Fritzinger, 2010, 2017). Moreover, humanized CVF does not induce a neutralizing antibody response, allowing for repeated administration (Ing et al., 2018).

#### Complement Depletion With Humanized CVF in a Murine Model of Myocardial Infarction Reperfusion Injury

**FIGURE 2:**
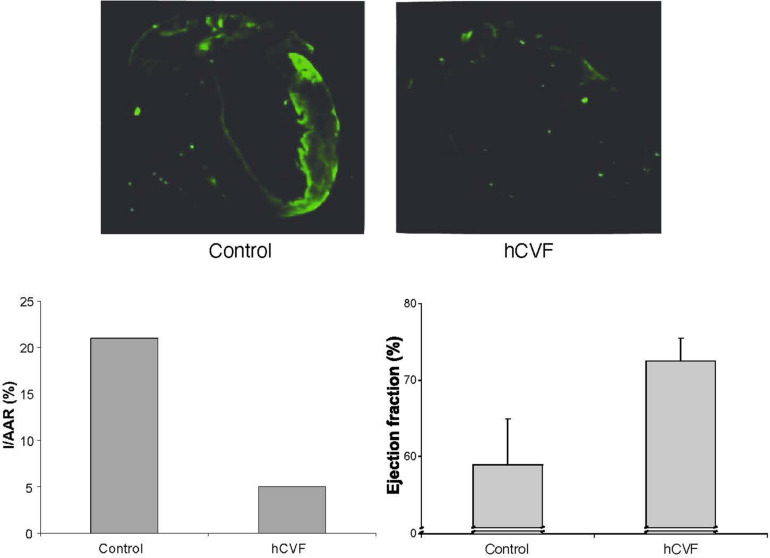
Effect of complement depletion with humanized CVF in a murine model of myocardial ischemia reperfusion injury. The upper panel shows immunohistochemical staining for C3 deposition. The lower left panel shows the size of the infarcted area as a percentage of the area at risk. The lower right panel shows the ejection fraction as a measure of left ventricular function (Gorsuch et al., 2009).

In a mouse model of myocardial infarction reperfusion injury, the mice were intubated, ventilated, and anesthesia was maintained with isoflurane. A suture was placed around the left anterior descending artery. After 30 min of ischemia, the ligation was loosened, and the myocardium reperfused for 4 h (Gorsuch et al., 2009). Both infarct size and area at risk were subsequently determined histologically with Evans Blue and triphenyl-tetrazolium chloride (TTC) (Walsh et al., 2005), demonstrating an approximately 75% reduction in size by treatment with humanized CVF (Figure 2; Gorsuch et al., 2009). C3 deposition was determined using an anti-mouse C3 antibody. The low extent of C3 deposition in animals depleted with humanized CVF reflects remaining C3. The ejection fraction was determined by transthoracic echocardiography (Gorsuch et al., 2009). Both parameters were dramatically improved by complement depletion with humanized CVF (Figure 2; Gorsuch et al., 2009). Collectively, these data demonstrate a significant reduction of myocardial injury by pathology, immunohistochemistry, and function.

In summary, complement depletion with humanized CVF appears to be a promising therapeutic approach for preventing or reducing myocardial ischemia reperfusion injury.

## Author Contributions

The author confirms being the sole contributor of this work and has approved it for publication.

## Conflict of Interest

The author has a financial interest in iC3 LLC of Sunnyvale, CA, a company that develops therapeutics for complement depletion.

## Figures and Tables

**FIGURE 3:**
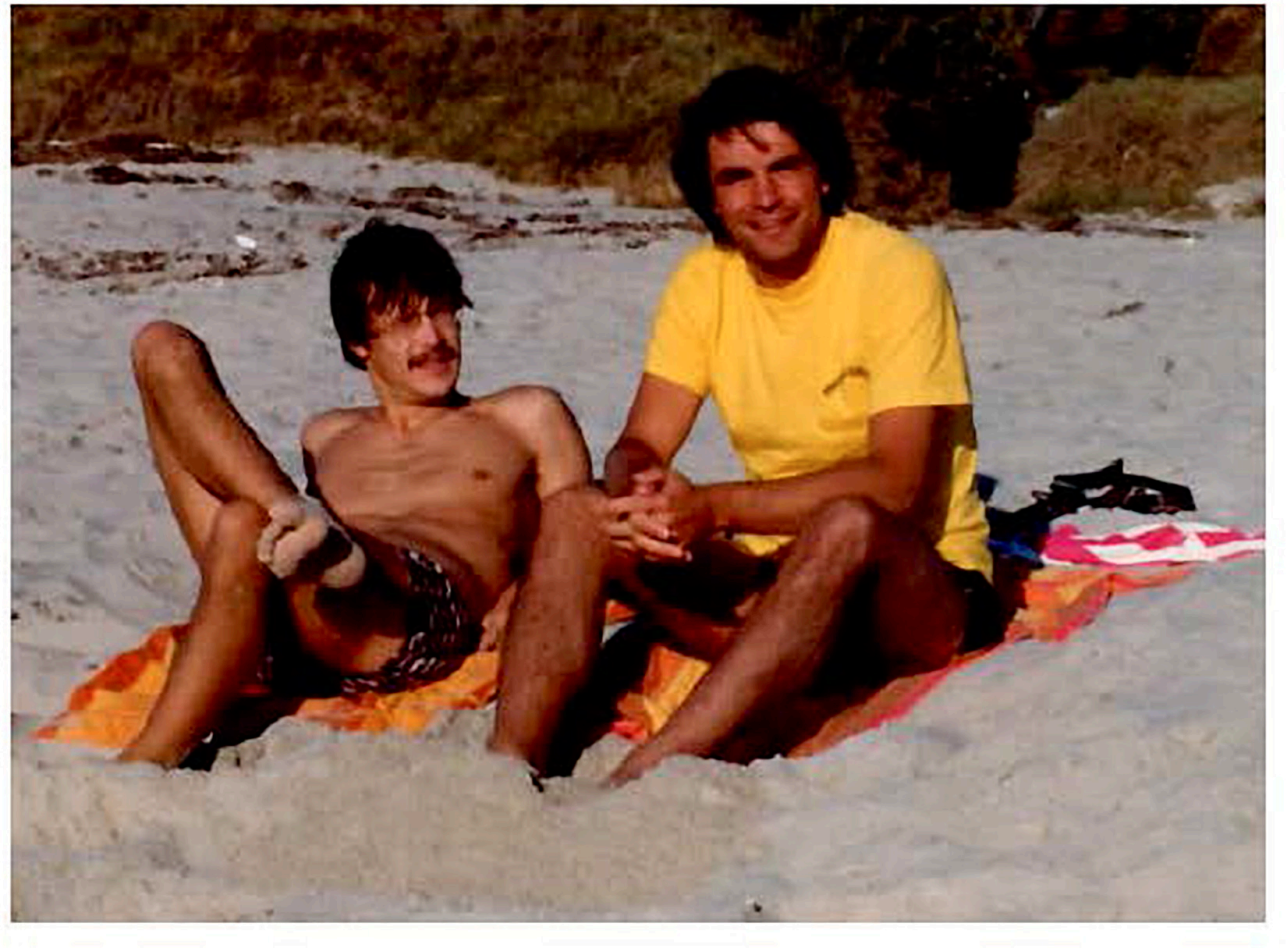
Dr. Klaus T. Preissner (left) and the author (right) at Windansea Beach, La Jolla, California (ca. 1980).

## Dedication

I am dedicating this article to Prof. Klaus T. Preissner, Professor Emeritus at the Justus-Liebig-University in Giessen, Germany, who has made many important contributions throughout his career to our knowledge of vascular biology. I had the pleasure and privilege of overlapping with Klaus as postdoctoral fellows from 1979 to 1982 at the Research Institute of Scripps Clinic, La Jolla, CA (Figure 3). We remained close personal and professional friends ever since.
